# Survival outcomes and prognostic nomogram for primary pulmonary MALT lymphoma and diffuse large B-cell lymphoma: A SEER database study

**DOI:** 10.17305/bb.2026.13944

**Published:** 2026-04-14

**Authors:** Jun Li, Yuan Feng, Huaying Zhang, Xin Zhou, Yunyan Tai

**Affiliations:** 1 Department of Oncology, Renmin Hospital, Hubei University of Medicine, Shiyan, Hubei, China; 2 Peking University Sixth Hospital, Peking University Institute of Mental Health, NHC Key Laboratory of Mental Health (Peking University), National Clinical Research Center for Mental Disorders (Peking University Sixth Hospital), Beijing, China; 3 Department of Respiratory and Critical Care Medicine, Renmin Hospital, Hubei University of Medicine, Shiyan, Hubei, China

**Keywords:** Pulmonary lymphoma, mucosa-associated lymphoid tissue lymphoma, diffuse large B-cell lymphoma, SEER database, survival analysis, nomogram

## Abstract

Primary pulmonary lymphoma is a rare extranodal malignancy, and robust population-based evidence regarding its treatment and outcomes remains limited. This study aimed to compare treatment patterns and survival outcomes between primary pulmonary mucosa-associated lymphoid tissue (MALT) lymphoma and primary pulmonary diffuse large B-cell lymphoma (DLBCL), as well as to develop an individualized prognostic model for pulmonary DLBCL. Patients diagnosed between 2010 and 2022 were identified from the Surveillance, Epidemiology, and End Results (SEER) database. Propensity score matching (PSM), Kaplan-Meier analysis, and Cox regression were employed to compare survival outcomes and identify prognostic factors. Additionally, a nomogram was constructed and internally validated for DLBCL. Among the 2,343 patients analyzed, 1,427 had MALT lymphoma and 916 had DLBCL. MALT lymphoma was more frequently managed with surgical intervention, while chemotherapy was more commonly utilized for DLBCL. After PSM, 441 matched pairs were analyzed, revealing that MALT lymphoma was significantly associated with better overall survival (OS) and cancer-specific survival (CSS) compared to DLBCL, with median OS of 131 months versus 68 months, respectively (*P <* 0.001). In patients with DLBCL, age ≥60 years emerged as an independent adverse prognostic factor, whereas surgical intervention and chemotherapy were independently associated with improved OS and CSS. The nomogram demonstrated moderate discrimination and good calibration in predicting 1-, 3-, and 5-year survival. These findings underscore that histological subtype is a critical determinant of prognosis in primary pulmonary lymphoma. The proposed nomogram may facilitate individualized prognostic assessments in pulmonary DLBCL.

## Introduction

Primary pulmonary lymphoma (PPL) is a rare form of extranodal lymphoma, representing approximately 0.4% of all pulmonary malignancies and 3%–4% of extranodal lymphomas [1, 2]. The histological spectrum of PPL is diverse, with mucosa-associated lymphoid tissue (MALT) lymphoma and diffuse large B-cell lymphoma (DLBCL) being the most prevalent subtypes, accounting for approximately 80%–90% of cases [3–5]. MALT lymphoma is characterized as a low-grade marginal zone lymphoma often associated with chronic antigenic stimulation, such as autoimmune diseases or chronic infections. It tends to exhibit slow progression, prolonged localized disease, and a favorable response to local treatment modalities [6–8]. In contrast, primary pulmonary DLBCL is an aggressive high-grade lymphoma characterized by rapid progression and a greater propensity for systemic dissemination [9, 10]. These fundamental biological differences necessitate distinct treatment strategies and result in markedly different prognostic outcomes between the two subtypes.

Despite the rarity of PPL, high-quality evidence to guide optimal management strategies is limited. Several critical clinical issues remain unresolved. First, most existing studies are derived from single-center retrospective cohorts or case reports, which restrict the generalizability of the findings. Second, low-grade and high-grade pulmonary lymphomas may present with similar radiological or clinical features at initial diagnosis, yet their treatment strategies and prognoses differ significantly, underscoring the importance of accurate classification. Third, the optimal management strategy for early-stage pulmonary MALT lymphoma—whether surgery, radiotherapy, or observation—remains contentious due to the absence of large comparative studies [11]. For pulmonary DLBCL, while rituximab, cyclophosphamide, doxorubicin, vincristine, and prednisone (R-CHOP)-based chemotherapy is regarded as the standard treatment, the roles of surgery and radiotherapy, particularly in cases of localized disease, remain uncertain [12, 13]. Finally, large population-based studies evaluating long-term survival outcomes and prognostic factors across these two major histological subtypes are still limited.

The Surveillance, Epidemiology, and End Results (SEER) database provides a valuable resource for studying rare malignancies due to its extensive population coverage and long-term follow-up data. However, population-based comparative analyses between pulmonary MALT lymphoma and pulmonary DLBCL remain scarce, and prognostic models specifically designed for pulmonary DLBCL are lacking. Therefore, this study aims to compare treatment patterns and survival outcomes between pulmonary MALT lymphoma and pulmonary DLBCL using the SEER database, as well as to develop and internally validate a prognostic prediction model for pulmonary DLBCL. Unlike previous registry-based studies that primarily focused on survival analysis, this research will develop and validate a prognostic nomogram and assess its clinical utility, providing a more practical tool for individualized survival prediction.

## Materials and methods

### Data sources and patient selection

Data for this investigation were obtained from the SEER database of the National Cancer Institute. We utilized SEERStat version 9.4.2 to identify patients diagnosed with PPL between 2010 and 2022. The analysis period concluded in 2022, as this was the most recent year with complete incidence and survival data available in the SEER database at the time of data extraction. PPL was defined as lymphoma originating in the lung and bronchus, recorded as the primary tumor site in the SEER database. To minimize the inclusion of systemic lymphoma with secondary pulmonary involvement, only patients with the lung designated as the primary site of malignancy were included. We strictly limited the primary site to C34.0–C34.9 (lung and bronchi) to ensure that the lesion originated in the lung. Additionally, we restricted the International Classification of Diseases for Oncology, Third Edition (ICD-O-3) histology codes to 9699/3 (MALT lymphoma) and 9680/3 (DLBCL). Given the extreme rarity of PPL in clinical practice, we did not impose additional restrictions on the primary cancer sequence number or the method of diagnostic confirmation to maximize the sample size and ensure the reliability of subsequent statistical analyses. The SEERStat selection filters were thus defined as follows: (1) Primary Site: C34.0-C34.9 (Lung and Bronchus); (2) Histology (ICD-O-3): 9699/3 and 9680/3; (3) Year of diagnosis: 2010–2022; (4) Age ≥18 years; (5) Known survival time ≥1 month; (6) Excluding cases identified by autopsy or death certificate only. Using these criteria, cases were operationally defined as PPL for this population-based analysis.

### Missing data processing

To ensure the accuracy of core baseline characteristics, we proactively excluded cases with missing or unknown information regarding race, marital status, and surgical history. Regarding American Joint Committee on Cancer (AJCC) staging, given the rarity of PPL, we did not exclude cases with unknown staging to maximize the sample size; instead, we categorized them uniformly as “Unknown” for analysis. In evaluating radiotherapy and chemotherapy, the SEER database’s objective recording mechanism combines “No treatment received” and “Treatment status unknown” into a single category labeled “No/Unknown.”

The SEER database does not provide information on treatment sequence or treatment line. Consequently, surgery, chemotherapy, and radiotherapy recorded in the SEER database were interpreted as treatments received during the initial course of therapy (first-course treatment), rather than specific treatment lines such as first-line or second-line therapy. Thus, treatment variables in this study reflect whether patients received these treatments as part of their management.

### Research variables and outcome indicators

The following variables were obtained from the SEER database: age, sex, race, marital status at diagnosis, presence of multiple primary malignancies, tumor site, AJCC stage, history of surgical intervention, radiation, chemotherapy, survival status, and survival duration. The primary outcomes of this research were overall survival (OS) and cancer-specific survival (CSS). OS is defined as the duration from the date of diagnosis to death from any cause, while CSS is defined as the duration from the date of diagnosis to death from tumor-related causes. In this study, staging refers to the SEER-derived AJCC stage rather than the Ann Arbor staging system traditionally used for lymphoma. All analyses involving stage were therefore based on the SEER AJCC staging variable.

### Statistical analysis

This study utilized the MatchIt package in R to calculate propensity scores using a logistic regression model, incorporating core variables such as sex, age, marital status, race, multiple cancers, primary site, AJCC stage, surgery, radiation, and chemotherapy as covariates in the matching model. The matching algorithm employed was 1:1 nearest neighbor matching (without replacement). To ensure high-quality matching and balance between groups, we set a stringent caliper value of 0.001 and used a standardized mean difference (SMD) of <0.1 between groups for each covariate post-matching as the criterion for evaluating balance. This approach allows for a more accurate assessment of survival differences and biological prognostic features between the two pathological subgroups (MALT and DLBCL), ultimately providing valuable prognostic assessments for patients with similar backgrounds in clinical practice. Survival curves were generated using the Kaplan-Meier method, and intergroup survival disparities were assessed through the log-rank test. Subsequently, patients with DLBCL were randomly allocated into a training set and a validation set in a 7:3 ratio. Independent prognostic factors were identified using univariate Cox regression analysis, with variables exhibiting *P <* 0.05 in univariate analysis entered into multivariate Cox regression to identify independent prognostic factors, which were subsequently used to construct the nomogram. The nomogram was developed using the training cohort only. Risk scores were calculated for each patient based on the nomogram. Model discrimination was evaluated using time-dependent receiver operating characteristic (ROC) curves, and the area under the curve (AUC) was calculated at 1, 3, and 5 years. Model calibration was assessed using calibration curves with 1,000 bootstrap resamples to compare predicted and observed survival probabilities. Decision curve analysis (DCA) was employed to evaluate the clinical utility of the model [14]. Regarding the division of high and low risk, we utilized the surv_cutpoint function from the R language survminer package, which is based on maximally selected rank statistics to determine the optimal cutoff value that maximally separates the survival curves. This standard, once established in the training set, was applied to both the training and validation sets to ensure logical rigor. Their survival was evaluated using Kaplan-Meier curves. A two-tailed *P* < 0.05 was considered statistically significant.

### Ethical statement

The SEER database is a publicly accessible resource that does not contain personally identifiable patient information, thereby negating the need for informed consent and ethical approval from an institutional review board.

## Results

### Patient baseline characteristics

This study included a total of 2,343 patients diagnosed with PPL, comprising 1,427 (60.9%) patients with MALT lymphoma and 916 (39.1%) patients with DLBCL. The patient selection process is illustrated in Figure 1. Male patients constituted 43.96% (1,030/2,343) of the overall cohort.

**Figure 1.**
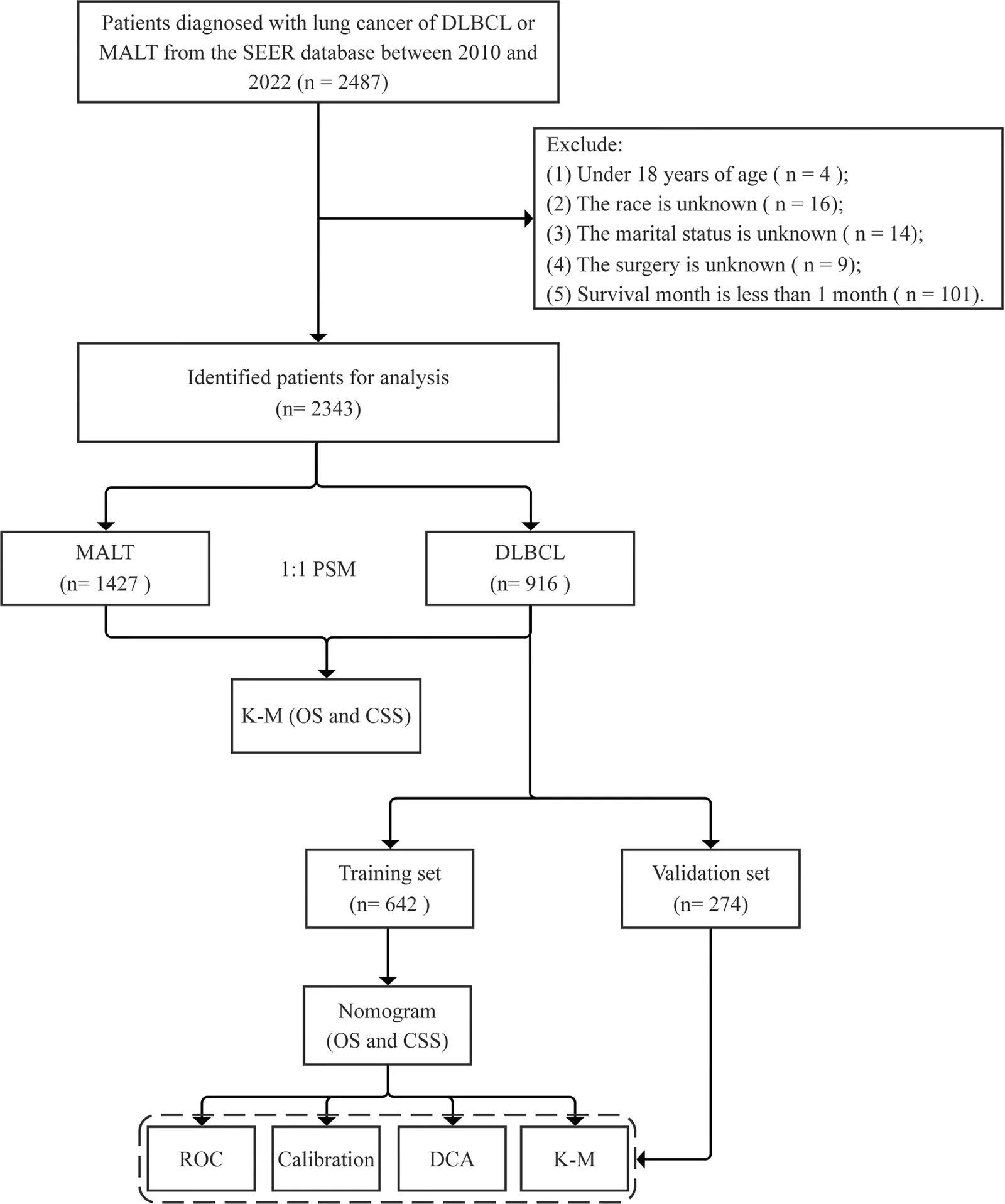
**Flowchart of data arrangement**. **Abbreviations:** CSS, cancer-specific survival; DCA, decision curve analysis; DLBCL, diffuse large B-cell lymphoma; K-M, Kaplan-Meier; MALT, mucosa-associated lymphoid tissue lymphoma; OS, overall survival; PSM, propensity score matching; ROC, receiver operating characteristic; SEER, Surveillance, Epidemiology, and End Results.

Prior to propensity score matching (PSM), baseline characteristics varied significantly between the two groups across several variables, particularly in stage distribution and treatment modalities. However, variables such as age, race, multiple primary cancers, and radiation therapy showed no statistically significant differences (Table S1). Patients with MALT lymphoma were more frequently diagnosed at early stages (stage I-II), accounting for 74.42% of cases, compared to 54.37% in the DLBCL group. In terms of treatment patterns, MALT lymphoma patients were more likely to undergo surgery (33.78% vs. 11.79%), whereas DLBCL patients were more likely to receive chemotherapy (77.73% vs. 22.07%). The proportion of patients receiving radiotherapy was similar in both groups (approximately 14%).

To mitigate baseline confounding and treatment selection bias, PSM was employed. After matching, each group consisted of 441 patients, with baseline characteristics well balanced between the two groups, showing no significant differences in age, sex, tumor location, stage, or treatment modalities (Table 1), thereby indicating successful matching.

**Table 1:** Comparison of baseline characteristics after PSM

**Variable**	**Overall**	**MALT**	**DLBCL**	***P***	**SMD**
n	882	441	441		
Sex, *n* (%)				>0.999	<0.001
Male	392 (44.44)	196 (44.44)	196 (44.44)		
Female	490 (55.56)	245 (55.56)	245 (55.56)		
Age, *n* (%)				0.340	0.070
<60	207 (23.47)	97 (22.00)	110 (24.94)		
≥60	675 (76.53)	344 (78.00)	331 (75.06)		
Marital status, *n* (%)				0.396	0.092
Unmarried	109 (12.36)	48 (10.88)	61 (13.83)		
Married	480 (54.42)	246 (55.78)	234 (53.06)		
SDW	293 (33.22)	147 (33.33)	146 (33.11)		
Race, *n* (%)				0.683	0.059
White	740 (83.90)	371 (84.13)	369 (83.67)		
Black	66 (7.48)	30 (6.80)	36 (8.16)		
Other	76 (8.62)	40 (9.07)	36 (8.16)		
Multiple cancers, *n* (%)				0.725	0.028
No	568 (64.40)	287 (65.08)	281 (63.72)		
Yes	314 (35.60)	154 (34.92)	160 (36.28)		
Primary site, *n* (%)				0.852	0.078
Main bronchus	18 (2.04)	9 (2.04)	9 (2.04)		
Upper lobe	247 (28.00)	122 (27.66)	125 (28.34)		
Middle lobe	59 (6.69)	28 (6.35)	31 (7.03)		
Lower lobe	249 (28.23)	132 (29.93)	117 (26.53)		
Others	309 (35.03)	150 (34.01)	159 (36.05)		
AJCC, *n* (%)				0.856	0.078
I	354 (40.14)	178 (40.36)	176 (39.91)		
II	173 (19.61)	92 (20.86)	81 (18.37)		
III	16 (1.81)	7 (1.59)	9 (2.04)		
IV	297 (33.67)	144 (32.65)	153 (34.69)		
Unknown	42 (4.76)	20 (4.54)	22 (4.99)		
Surgery, *n* (%)				0.383	0.065
No	758 (85.94)	384 (87.07)	374 (84.81)		
Yes	124 (14.06)	57 (12.93)	67 (15.19)		
Radiation, *n* (%)				0.919	0.014
No/Unknown	772 (87.53)	387 (87.76)	385 (87.30)		
Yes	110 (12.47)	54 (12.24)	56 (12.70)		
Chemotherapy, *n* (%)				0.734	0.027
No/Unknown	384 (43.54)	195 (44.22)	189 (42.86)		
Yes	498 (56.46)	246 (55.78)	252 (57.14)		

**Abbreviations:** AJCC, American Joint Committee on Cancer; DLBCL, diffuse large B-cell lymphoma; MALT, mucosa-associated lymphoid tissue lymphoma; PSM, propensity score matching; SDW, separated, divorced, and widowed; SMD, standardized mean difference.

### Survival analysis

Before PSM, the median follow-up duration for OS was 83 months (95% Confidence Interval [CI]: 77–89 months), and the median survival time was 74 months (95% CI: 62–89 months). The 1-year, 3-year, and 5-year OS rates for the entire cohort were 70.8%, 60.1%, and 53.7%, respectively. The median follow-up duration for CSS was 66 months (95% CI: 61–71), with the median survival time not yet reached. The 1-year, 3-year, and 5-year CSS rates were 76.8%, 68.7%, and 65.4%, respectively. Kaplan–Meier analysis across all groups demonstrated significantly superior OS and CSS in patients with MALT lymphoma compared to those with DLBCL. The median OS was not reached in the MALT cohort, while it was 74 months (95% CI: 62–89) in the DLBCL cohort; median CSS was not reached in either group (Figure S1). In the PSM cohort, MALT lymphoma patients continued to exhibit superior OS and CSS, with a median OS of 131 months (95% CI: 107–not reached) compared to 68 months (95% CI: 41–86) in the DLBCL group; median CSS remained not reached in both cohorts, as illustrated in Figure 2.

**Figure 2.**
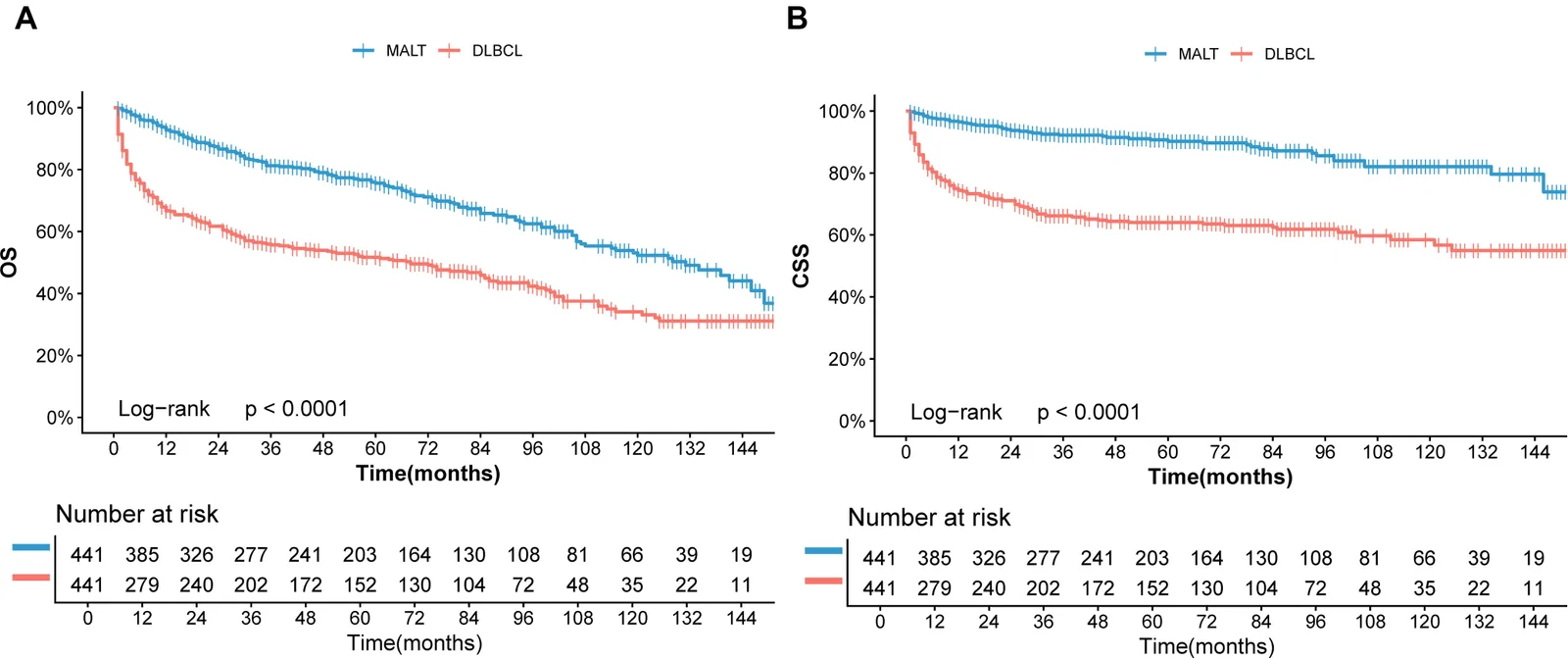
**Kaplan–Meier curves for overall survival and cancer-specific survival after propensity score matching.** (A) Overall survival in the matched cohort (441 patients per group) remained significantly better in patients with primary pulmonary MALT lymphoma than in those with DLBCL (log-rank *P <* 0.0001). Median overall survival was 131 months (95% CI: 107–not reached) in the MALT group and 68 months (95% CI: 41–86) in the DLBCL group. (B) Cancer-specific survival in the matched cohort was also significantly better in the MALT group than in the DLBCL group (log-rank *P <* 0.0001), whereas median cancer-specific survival was not reached in either group. Tick marks indicate censored observations, and the numbers at risk are shown below each plot. **Abbreviations:** CI, confidence interval; CSS, cancer-specific survival; DLBCL, diffuse large B-cell lymphoma; MALT, mucosa-associated lymphoid tissue; OS, overall survival; PSM, propensity score matching.

### Prognostic model

#### Baseline table for training and validation sets

The study encompassed 916 individuals with DLBCL, who were randomly assigned to a training cohort (642 cases) and a validation cohort (274 instances). The two groups exhibited no significant statistical differences in demographic characteristics, tumor features, or treatment strategies. Only chemotherapy variables showed significant differences, indicating overall good balance between the groups (Table 2).

**Table 2:** Comparative analysis of baseline characteristics across training and validation sets

**Variables**	**Total (*n* ═ 916)**	**Training set** **(n =** **642)**	**Validation set** **(*n* ═ 274)**	***P***
Sex, *n* (%)				0.933
Male	441 (48.14)	308 (47.98)	133 (48.54)	
Female	475 (51.86)	334 (52.02)	141 (51.46)	
Age, *n* (%)				0.538
<60	230 (25.11)	157 (24.45)	73 (26.64)	
≥60	686 (74.89)	485 (75.55)	201 (73.36)	
Marital status, *n* (%)				0.697
Unmarried	144 (15.72)	103 (16.04)	41 (14.96)	
Married	502 (54.8)	355 (55.3)	147 (53.65)	
SDW	270 (29.48)	184 (28.66)	86 (31.39)	
Race, *n* (%)				0.164
White	784 (85.59)	554 (86.29)	230 (83.94)	
Black	58 (6.33)	43 (6.7)	15 (5.47)	
Other	74 (8.08)	45 (7.01)	29 (10.58)	
Multiple cancers, *n* (%)			0.530	
No	603 (65.83)	418 (65.11)	185 (67.52)	
Yes	313 (34.17)	224 (34.89)	89 (32.48)	
Primary site, *n* (%)				0.411
Main bronchus	37 (4.04)	23 (3.58)	14 (5.11)	
Upper lobe	249 (27.18)	180 (28.04)	69 (25.18)	
Middle lobe	48 (5.24)	36 (5.61)	12 (4.38)	
Lower lobe	266 (29.04)	191 (29.75)	75 (27.37)	
Others	316 (34.5)	212 (33.02)	104 (37.96)	
AJCC, *n* (%)				0.785
I	257 (28.06)	174 (27.1)	83 (30.29)	
II	241 (26.31)	169 (26.32)	72 (26.28)	
III	44 (4.8)	32 (4.98)	12 (4.38)	
IV	343 (37.45)	243 (37.85)	100 (36.5)	
Unknown	31 (3.38)	24 (3.74)	7 (2.55)	
Surgery, *n* (%)				0.789
No	808 (88.21)	568 (88.47)	240 (87.59)	
Yes	108 (11.79)	74 (11.53)	34 (12.41)	
Radiation, *n* (%)				0.073
No/Unknown	785 (85.7)	541 (84.27)	244 (89.05)	
Yes	131 (14.3)	101 (15.73)	30 (10.95)	
Chemotherapy, *n* (%)				0.007
No/Unknown	204 (22.27)	159 (24.77)	45 (16.42)	
Yes	712 (77.73)	483 (75.23)	229 (83.58)	

**Abbreviations:** AJCC, American Joint Committee on Cancer; SDW, separated, divorced, and widowed.

#### Univariate and multivariate Cox regression for OS and CSS

Results from univariate and multivariate Cox regression analyses (Table 3) indicate that age ≥60 years (hazard ratio [HR]=2.632) is an independent risk factor, while surgery (HR=0.454) and chemotherapy (HR=0.501) serve as independent protective factors for OS. Regarding CSS (Table 4), age ≥60 years (HR=1.875) is also an independent risk factor, with surgery (HR=0.488) and chemotherapy (HR=0.450) identified as independent protective factors.

**Table 3:** Univariate and multivariate Cox regression analysis of OS

**Variable**	**Univariate**			**Multivariate**		
	**HR**	**95%CI**	***P***	**HR**	**95%CI**	***P***
*Sex*						
Male	Ref					
Female	0.923	0.741–1.149	0.471			
*Age*						
<60	Ref			Ref		
≥60	2.791	2.032–3.834	<0.001	2.632	1.894–3.657	<0.001
*Marital*						
Unmarried	Ref			Ref		
Married	0.993	0.714–1.380	0.966	0.746	0.533–1.044	0.088
SDW	1.704	1.212–2.394	0.002	1.143	0.805–1.622	0.455
*Race*						
White	Ref					
Black	1.148	0.744–1.773	0.533			
Other	1.012	0.661–1.549	0.956			
*Multiple cancers*						
No	Ref			Ref		
Yes	1.332	1.065–1.665	0.012	1.080	0.862–1.355	0.503
*Primary site*						
Main bronchus	Ref					
Upper lobe	1.001	0.534–1.879	0.997			
Middle lobe	0.808	0.367–1.781	0.597			
Lower lobe	1.041	0.556–1.949	0.899			
Others	1.468	0.793–2.720	0.222			
*AJCC*						
I	Ref					
II	0.931	0.688–1.260	0.645			
III	0.819	0.474–1.416	0.474			
IV	0.985	0.749–1.294	0.912			
Unknown	1.217	0.692–2.138	0.495			
*Surgery*						
No	Ref			Ref		
Yes	0.524	0.350–0.782	0.002	0.454	0.304–0.680	<0.001
*Radiation*						
No/Unknown	Ref					
Yes	0.752	0.549–1.031	0.077			
*Chemotherapy*						
No/Unknown	Ref			Ref		
Yes	0.452	0.357–0.571	<0.001	0.501	0.394–0.637	<0.001

**Abbreviations:** AJCC, American Joint Committee on Cancer; CI, confidence interval; HR, hazard ratio; OS, overall survival; Ref, reference; SDW, separated, divorced, and widowed.

**Table 4:** Univariate and multivariate Cox regression analysis of CSS

**Variable**	**Univariate**			**Multivariate**		
	**HR**	**95% CI**	***P***	**HR**	**95%CI**	***P***
*Sex*						
Male	Ref					
Female	0.924	0.699–1.221	0.579			
*Age*						
<60	Ref			Ref		
≥60	2.013	1.388–2.918	<0.001	1.875	1.290–2.724	<0.001
*Marital*						
Unmarried	Ref					
Married	0.811	0.545–1.208	0.303			
SDW	1.409	0.934–2.124	0.102			
*Race*						
White	Ref					
Black	1.441	0.875–2.374	0.151			
Other	0.941	0.535–1.654	0.832			
*Multiple cancers*						
No	Ref					
Yes	1.280	0.963–1.702	0.089			
*Primary site*						
Main bronchus	Ref					
Upper lobe	1.038	0.472–2.281	0.926			
Middle lobe	0.959	0.365–2.519	0.932			
Lower lobe	0.914	0.414–2.018	0.824			
Others	1.413	0.652–3.063	0.381			
*AJCC*						
I	Ref					
II	1.073	0.717–1.604	0.733			
III	1.185	0.615–2.286	0.612			
IV	1.307	0.915–1.869	0.141			
Unknown	1.291	0.610–2.733	0.504			
*Surgery*						
No	Ref			Ref		
Yes	0.546	0.327–0.911	0.021	0.488	0.292–0.816	0.006
*Radiation*						
No/Unknown	Ref					
Yes	0.740	0.493–1.111	0.147			
*Chemotherapy*						
No/Unknown	Ref			Ref		
Yes	0.439	0.327–0.590	<0.001	0.450	0.334–0.605	<0.001

**Abbreviations:** AJCC, American Joint Committee on Cancer; CI, confidence interval; CSS, cancer-specific survival; HR, hazard ratio; Ref, reference; SDW, separated, divorced, and widowed.

#### Nomogram

The nomogram developed in this study incorporates key prognostic variables such as age, surgical intervention, and chemotherapy. By summing the corresponding scores for each variable, a total patient score is generated, enabling the estimation of individualized survival probabilities. This framework supports the formulation of personalized management strategies in clinical practice, as depicted in Figure 3.

**Figure 3.**
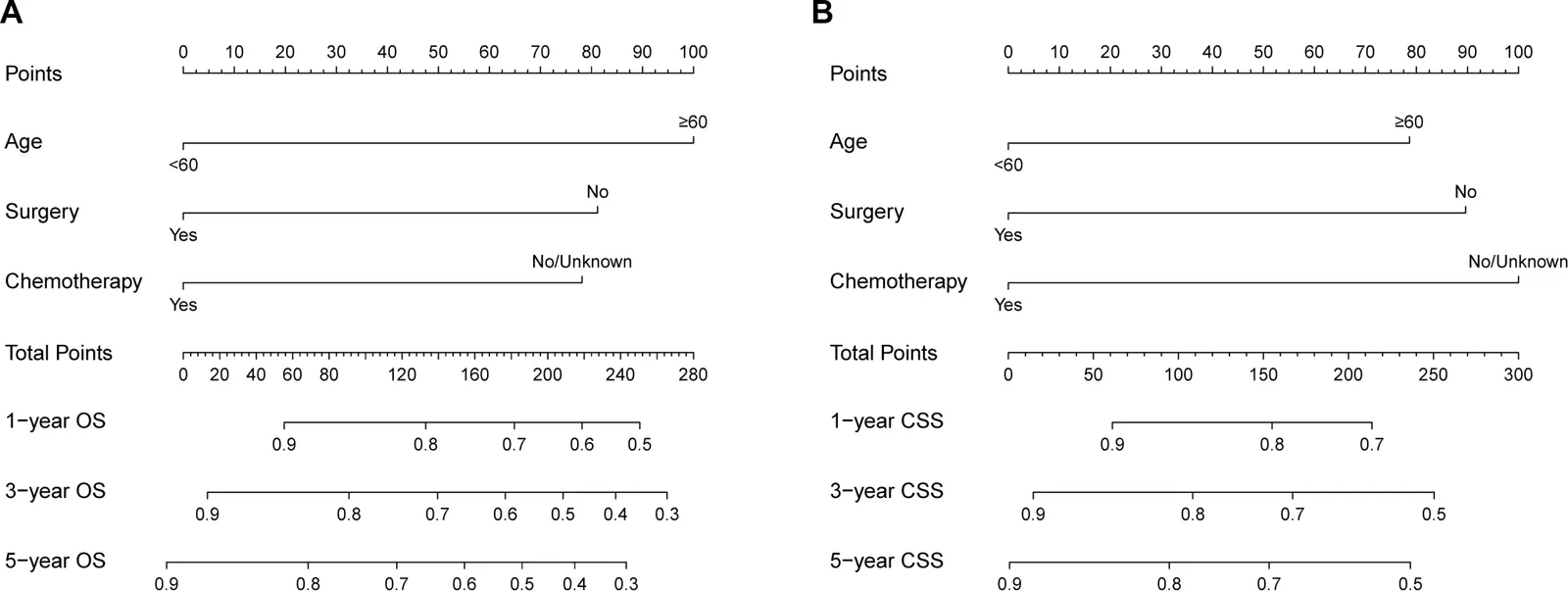
**Nomograms for individualized prediction of overall survival and cancer-specific survival in primary pulmonary diffuse large B-cell lymphoma.** (A) Nomogram for estimating 1-, 3-, and 5-year overall survival. (B) Nomogram for estimating 1-, 3-, and 5-year cancer-specific survival. Both nomograms incorporate age, surgery, and chemotherapy as independent prognostic variables. For each patient, points are assigned for each variable according to the corresponding axis; the sum of these values generates the total points, which are then projected onto the lower scales to estimate the predicted probability of overall survival or cancer-specific survival at 1, 3, and 5 years. These models provide a practical tool for individualized prognostic assessment and may support risk stratification in clinical practice. **Abbreviations:** CSS, cancer-specific survival; DLBCL, diffuse large B-cell lymphoma; OS, overall survival.

#### Model performance evaluation

The predictive efficacy of the developed models was rigorously assessed utilizing calibration curves, ROC curves, and DCA. The calibration curves demonstrated a strong correspondence between anticipated and actual survival rates at each time point, indicating exceptional calibration and stability of the OS and CSS models (Figure 4). ROC curve analysis revealed that the predictive models exhibited robust performance in distinguishing patients with varying prognostic risks, maintaining consistent predictive efficacy across both training and validation datasets. The AUC values for OS in the training set at 1, 3, and 5 years were 0.704, 0.677, and 0.681, respectively; the validation set exhibited AUC values of 0.622, 0.662, and 0.693, respectively. The training set for CSS yielded AUC values of 0.703, 0.663, and 0.653 at 1, 3, and 5 years, respectively; the validation set showed comparable values of 0.646, 0.660, and 0.686 (Figure 5). Furthermore, DCA results indicated that the nomogram for forecasting OS and CSS provided a higher net benefit compared to traditional staging systems, suggesting superior practicality and effectiveness in clinical decision-making (Figure 6).

**Figure 4.**
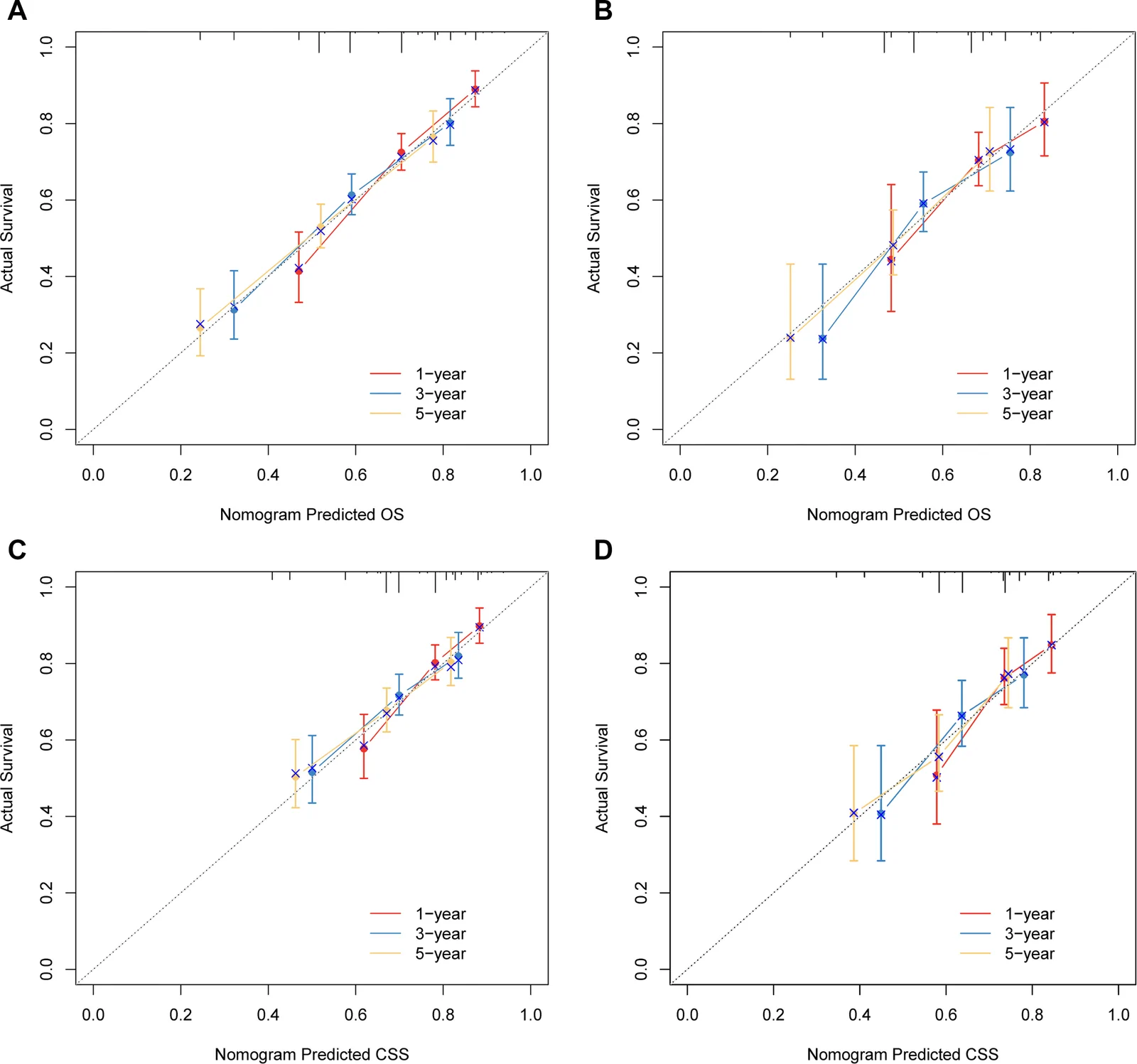
**Calibration plots of the nomogram for predicting 1-, 3-, and 5-year overall survival and cancer-specific survival in primary pulmonary diffuse large B-cell lymphoma.** (A) Calibration curves for overall survival in the training cohort. (B) Calibration curves for overall survival in the validation cohort. (C) Calibration curves for cancer-specific survival in the training cohort. (D) Calibration curves for cancer-specific survival in the validation cohort. The x-axis represents nomogram-predicted survival probability, and the y-axis represents observed survival probability. The 45-degree reference line indicates perfect agreement between predicted and observed outcomes; calibration curves lying closer to this line reflect better model calibration. Across both the training and validation cohorts, the 1-, 3-, and 5-year curves showed good agreement with the reference line, indicating satisfactory calibration and stability of the nomogram. **Abbreviations:** CSS, cancer-specific survival; DLBCL, diffuse large B-cell lymphoma; OS, overall survival.

**Figure 5.**
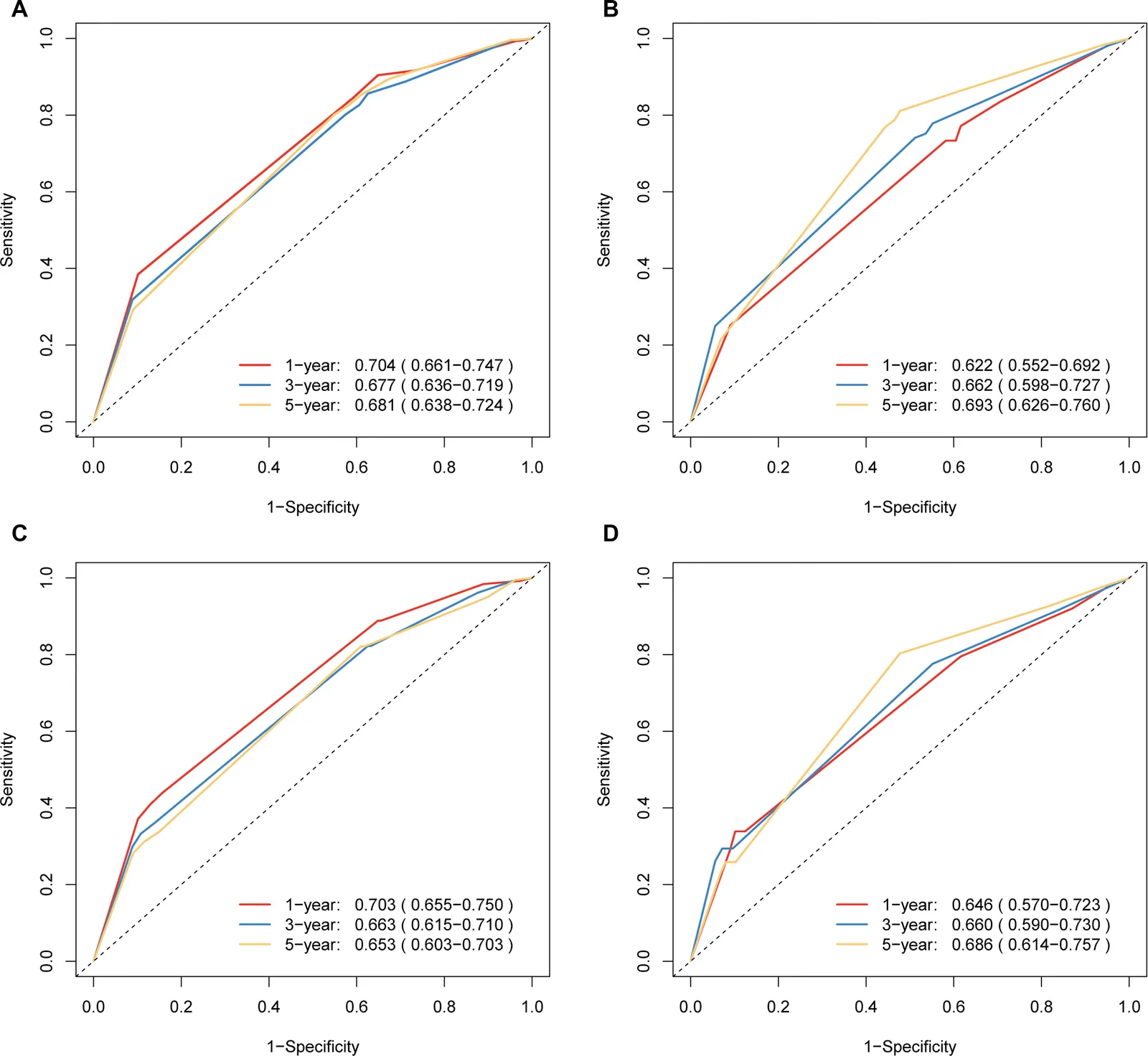
**Time-dependent ROC curves of the nomogram for predicting overall survival and cancer-specific survival in patients with primary pulmonary diffuse large B-cell lymphoma.** (A) ROC curves for 1-, 3-, and 5-year overall survival in the training cohort, with AUC values of 0.704 (95% CI: 0.661–0.747), 0.677 (95% CI: 0.636–0.719), and 0.681 (95% CI: 0.639–0.724), respectively. (B) ROC curves for 1-, 3-, and 5-year overall survival in the validation cohort, with AUC values of 0.622 (95% CI: 0.552–0.692), 0.662 (95% CI: 0.598–0.727), and 0.693 (95% CI: 0.620–0.766), respectively. (C) ROC curves for 1-, 3-, and 5-year cancer-specific survival in the training cohort, with AUC values of 0.703 (95% CI: 0.655–0.750), 0.663 (95% CI: 0.615–0.710), and 0.653 (95% CI: 0.603–0.703), respectively. (D) ROC curves for 1-, 3-, and 5-year cancer-specific survival in the validation cohort, with AUC values of 0.646 (95% CI: 0.570–0.723), 0.660 (95% CI: 0.590–0.730), and 0.686 (95% CI: 0.614–0.757), respectively. The diagonal dashed line represents the reference line for no discrimination. Overall, the nomogram showed moderate and relatively stable discriminatory performance across both the training and validation cohorts. **Abbreviations:** AUC, area under the curve; CI, confidence interval; CSS, cancer-specific survival; OS, overall survival; ROC, receiver operating characteristic.

**Figure 6.**
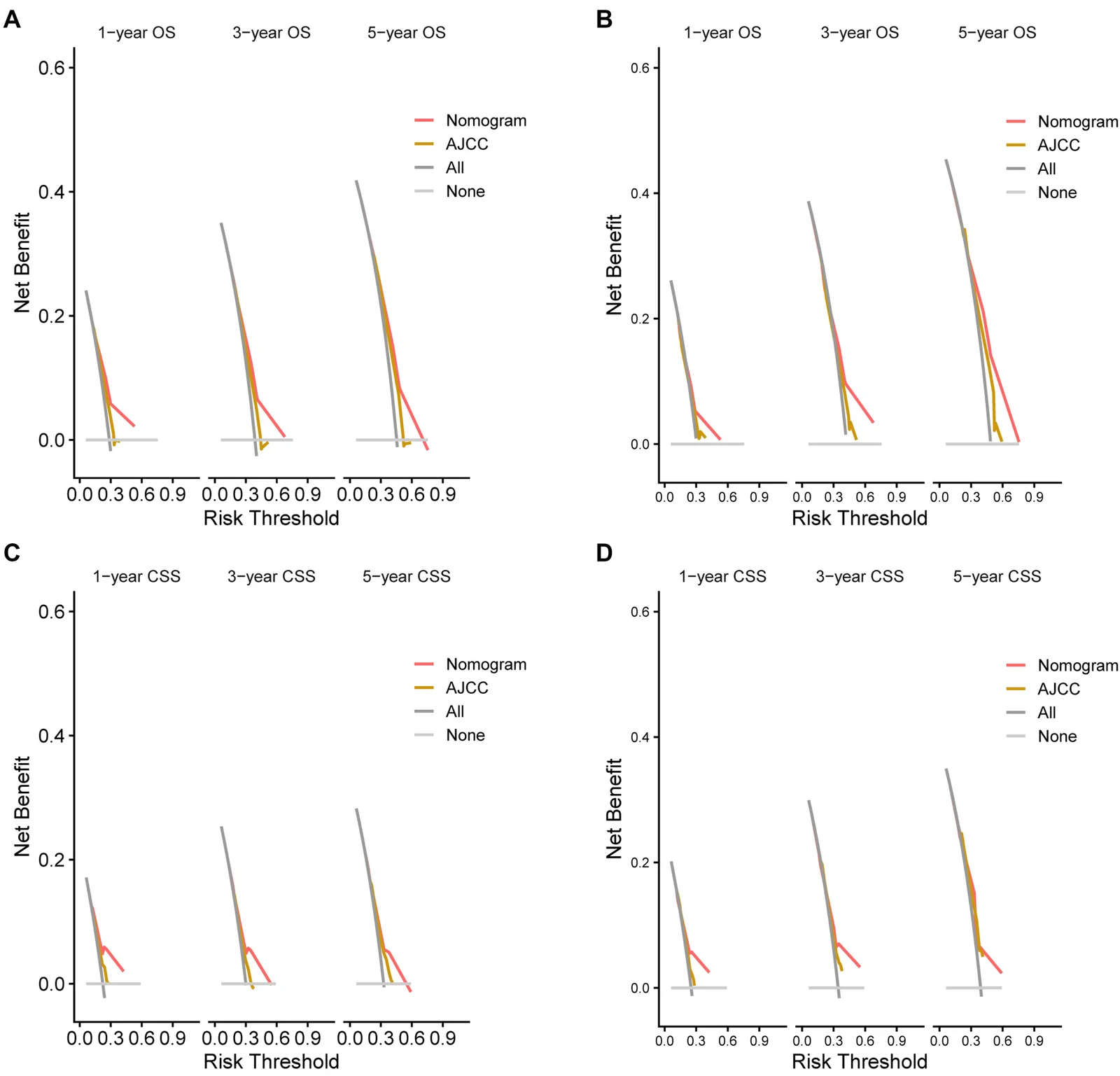
**DCA comparing the nomogram with the American Joint Committee on Cancer staging system for predicting 1-, 3-, and 5-year overall survival and cancer-specific survival.** (A) DCA for overall survival in the training cohort. (B) DCA for overall survival in the validation cohort. (C) DCA for cancer-specific survival in the training cohort. (D) DCA for cancer-specific survival in the validation cohort. In each panel, the x-axis represents the threshold probability and the y-axis represents net benefit. The red curve indicates the nomogram, the yellow curve indicates the American Joint Committee on Cancer staging system, and the gray lines represent the treat-all and treat-none strategies. Across both training and validation cohorts, the nomogram generally provided greater net benefit than the staging system over clinically relevant threshold ranges for predicting 1-, 3-, and 5-year overall survival and cancer-specific survival, supporting its potential clinical utility. **Abbreviations:** AJCC, American Joint Committee on Cancer; CSS, cancer-specific survival; DCA, decision curve analysis; OS, overall survival.

#### High- and low-risk Kaplan–Meier curves

Total scores were calculated for each patient using the nomogram, with optimal cutoff values established in the training set through maximally selected rank statistics. Separate nomograms were developed for OS and CSS, resulting in differing cutoff values and corresponding risk group distributions. For the OS model, the cutoff value was 100.00. In the training cohort, 141 patients were classified as low risk and 501 as high risk; in the validation cohort, 64 patients were classified as low risk and 210 as high risk. Kaplan-Meier analysis revealed that the high-risk group exhibited significantly worse OS compared to the low-risk group in both the training and validation cohorts (both log-rank *P <* 0.0001; Figure 7A and B). For the CSS model, the cutoff value was 189.65. In the training cohort, 504 patients were classified as low risk and 138 as high risk; in the validation cohort, 233 patients were classified as low risk and 41 as high risk. Similarly, patients in the high-risk group demonstrated significantly worse CSS compared to those in the low-risk group in both cohorts (both log-rank *P <* 0.0001; Figure 7C and D). The totals in each panel were consistent with the original DLBCL cohort distribution, comprising 642 patients in the training set and 274 patients in the validation set.

**Figure 7.**
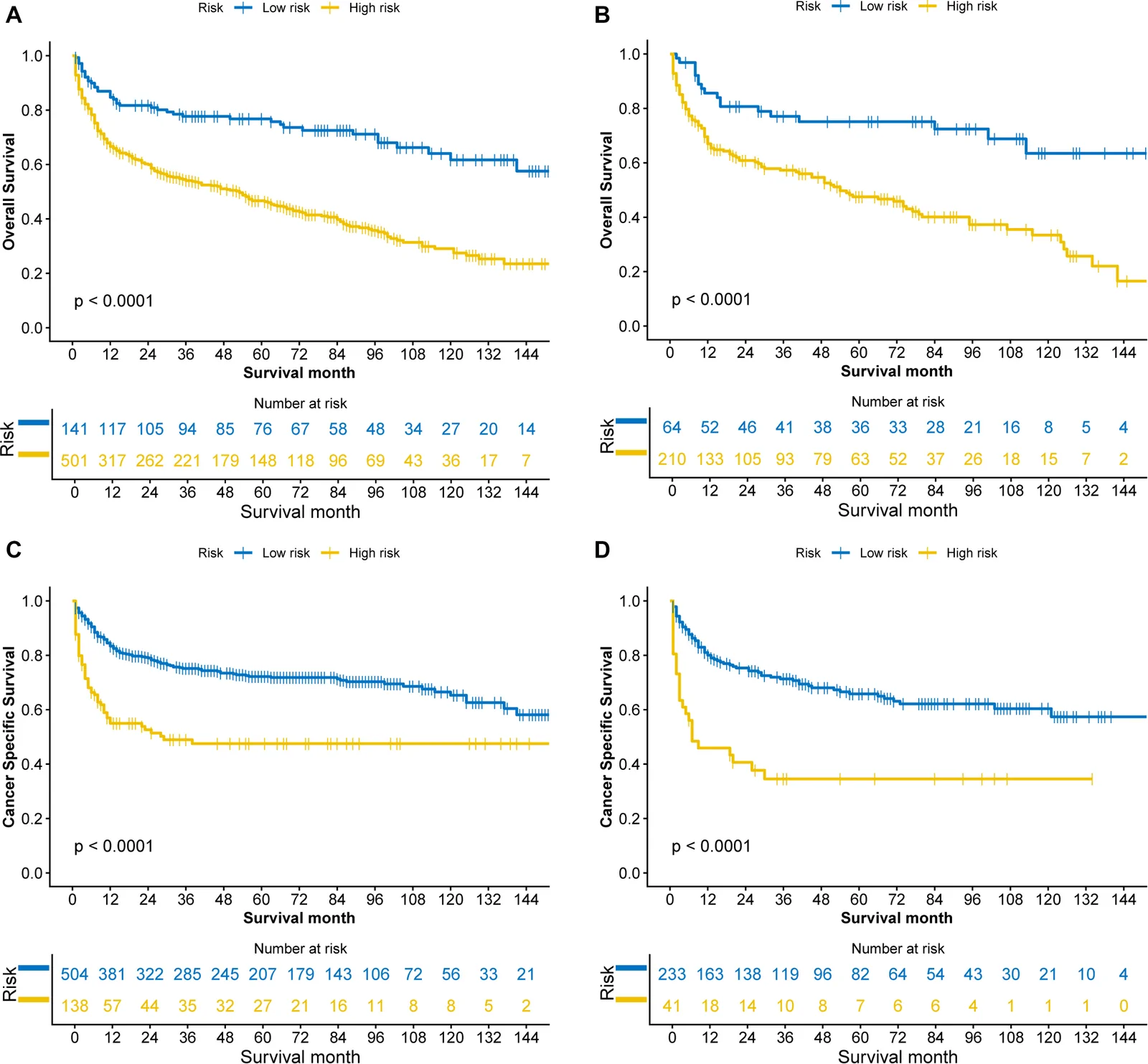
**Kaplan–Meier survival curves for nomogram-based risk stratification in primary pulmonary diffuse large B-cell lymphoma.** Patients were stratified into low-risk and high-risk groups according to nomogram-derived total scores using cutoff values determined in the training set by maximally selected rank statistics. (A) Overall survival in the training cohort, using an overall survival cutoff of 100.00 (low risk, *n* ═ 141; high risk, *n* ═ 501). (B) Overall survival in the validation cohort, using the same overall survival cutoff (low risk, *n* ═ 64; high risk, *n* ═ 210). (C) Cancer-specific survival in the training cohort, using a cancer-specific survival cutoff of 189.65 (low risk, *n* ═ 504; high risk, *n* ═ 138). (D) Cancer-specific survival in the validation cohort, using the same cancer-specific survival cutoff (low risk, *n* ═ 233; high risk, *n* ═ 41). In both the training and validation cohorts, the high-risk group had significantly worse overall survival and cancer-specific survival than the low-risk group (all log-rank *P <* 0.0001). Tick marks indicate censored observations, and the numbers at risk are shown below each plot. The totals in each panel correspond to the predefined diffuse large B-cell lymphoma cohort split, with 642 patients in the training set and 274 patients in the validation set. **Abbreviations:** CSS, cancer-specific survival; DLBCL, diffuse large B-cell lymphoma; OS, overall survival.

## Discussion

### Pathological subtype as the primary prognostic determinant

In this study, AJCC stage was not identified as an independent prognostic factor in multivariate analysis. This finding may be attributed to the limited applicability of the AJCC staging system in PPL, which frequently manifests as localized extranodal disease. Consequently, clinical factors such as age and treatment variables may exert a more significant influence on prognosis than traditional staging in this specific disease context. The study revealed considerable disparities in real-world treatment approaches between two lymphoma types: the proportion of patients receiving chemotherapy in the DLBCL cohort was significantly higher than in the MALT cohort (77.73% vs. 22.07%), whereas the percentage of patients undergoing surgical intervention was greater in the MALT cohort (33.78% vs. 11.79%). These findings align with current clinical practices. The most critical discovery of this research is that after PSM, the two patient groups achieved balance regarding all confounding factors, yet a substantial survival disparity persisted (median OS: 131 months vs. 68 months). This indicates that the pathological diagnosis of “MALT lymphoma vs. DLBCL” serves as a more crucial survival predictor than conventional prognostic markers such as stage, age, and treatment [8, 15]. The underlying cause is rooted in their distinct biological characteristics: MALT lymphoma, classified as a low-grade lymphoma, is often associated with chronic stimulation by specific antigens (e.g., Helicobacter pylori in gastric MALT) and exhibits slow cell proliferation [13, 16]. MALT lymphoma typically involves gene translocations such as mucosa-associated lymphoid tissue lymphoma translocation protein 1 (MALT1) and B-cell lymphoma/leukemia 10 (BCL10), leading to persistent activation of the nuclear factor kappa-light-chain-enhancer of activated B cells (NF-κB) pathway, albeit in a relatively “mild” manner [17]. In contrast, DLBCL is highly aggressive and characterized by significant molecular heterogeneity, including high-risk subtypes such as MYC proto-oncogene (MYC) and B-cell lymphoma 2 (BCL-2)/B-cell lymphoma 6 (BCL-6) rearrangements, with substantial proliferation and dissemination potential, even in early stages [15, 18, 19]. In particular, DLBCL originating from the lungs has a more unstable genome, and non-germinal center B-cell-like (GCB) subtypes typically have worse prognoses [20]. Recent advancements in medical treatments, such as CD19 chimeric antigen receptor T-cell (CAR-T) therapy for DLBCL, bispecific antibodies like glofitamab, and improved chemotherapy regimens, including dose-adjusted etoposide, prednisone, vincristine, cyclophosphamide, doxorubicin, and rituximab (DA-EPOCH-R), have enhanced outcomes for specific high-risk patients. However, these innovations have not significantly changed the overall aggressive course of the disease [17, 21, 22]. In contrast, local radiation, surgery, or even a “watchful waiting” approach for certain asymptomatic patients with pulmonary MALT lymphoma can lead to long-term survival, highlighting the disease’s more benign nature [23, 24]. By incorporating treatment variables into the propensity score model, the post-PSM survival comparison reflects survival differences primarily attributable to pathological subtype rather than treatment selection, thereby isolating the biological and prognostic distinctions between MALT lymphoma and DLBCL.

While age and treatment variables have been recognized as prognostic factors in lymphoma, this study makes several novel contributions. First, PPL is a rare disease, and large population-based studies comparing pulmonary MALT lymphoma and pulmonary DLBCL are scarce. Second, this study utilized a large SEER-based cohort and applied PSM to minimize baseline differences, enabling a more balanced comparison of survival outcomes between the two histological subtypes. Third, we developed and internally validated a prognostic nomogram specifically for pulmonary DLBCL, which may facilitate individualized risk assessment in this rare disease entity. Therefore, the novelty of this study resides not in the identification of entirely new prognostic factors but in providing population-based comparative evidence and a disease-specific prognostic model for PPL.

### Hierarchical interpretation of prognostic factors

This study meticulously examined the prognostic variables of patients with primary DLBCL of the lung using multivariate Cox regression. Age over 60 years emerged as an independent adverse predictive factor, a finding consistent with patterns observed in solid tumor and lymphoma studies, reflecting the combined effects of diminished tolerance to intensive therapy, multiple comorbidities, and declining immune function in the elderly [25]. This underscores the critical need for comprehensive geriatric assessments in elderly patients to balance treatment intensity with tolerance [26]. In the multivariate analysis, surgery was identified as a significant protective factor for OS and CSS. This suggests that surgical resection, whether diagnostic or radical, may confer a survival benefit for selected patients with limited-stage lung DLBCL by reducing tumor burden, eliminating drug-resistant clones, or synergizing with subsequent chemotherapy [27]. Chemotherapy emerged as the strongest protective factor, reinforcing the foundational role of chemotherapy-based systemic therapy in managing lung DLBCL [28]. Although the database used in this study did not specify whether rituximab was included, this finding aligns with the nature of DLBCL as a systemic disease characterized by high sensitivity to chemotherapy [12, 29, 30]. However, a notable finding was that staging did not demonstrate significant prognostic value in either univariate or multivariate analyses (*P >* 0.05). This challenges conventional wisdom and may indicate that the inherently aggressive nature of pulmonary DLBCL presents a significant risk of systemic spread even in localized stages, thereby diminishing the prognostic discriminative power of initial anatomical extent. Additionally, the dominant impact of systemic chemotherapy may obscure the influence of staging; when most patients receive chemotherapy, the effect of initial tumor burden on ultimate outcomes is less pronounced. Furthermore, the AJCC staging system may have limited applicability to primary pulmonary DLBCL, a specific extranodal lesion, and may require modification to incorporate indicators such as tumor size and lactate dehydrogenase levels. This finding serves as a cautionary note that treatment decisions for lung DLBCL should not overly rely on staging but should prioritize biological indicators (such as molecular subtyping) that reflect disease invasiveness and treatment tolerance. Meanwhile, the persistently poor survival prognosis of the DLBCL group after PSM suggests that the current standard chemotherapy regimen may have reached its “ceiling of efficacy,” underscoring the urgent need to explore the application of new targeted therapies, antibody-drug conjugates, or intensified treatment strategies for this patient population [31].

### Construction and clinical value of prognostic models

A nomogram based on independent prognostic criteria provides an intuitive tool for determining individualized survival probabilities. The model demonstrates robust discrimination and strong calibration, and DCA further highlights its potential for clinical application. However, the AUC value not exceeding 0.75 objectively reflects the limitations of the current model: it incorporates only a limited number of clinical variables, such as age, surgery, and chemotherapy, while lacking international prognostic indices and key molecular pathological features (e.g., cell origin and dual expression/triple hit status). This points to a critical opportunity for future research: a truly effective prognostic prediction model must be multidimensionally integrated, combining clinical characteristics, treatment details, and radiomic data, thereby facilitating a transition from “population statistics” to “individual prediction.”

### Limitations and future directions of the research

This research is subject to several limitations. First, the SEER database lacks critical information, including specific chemotherapy agents (notably rituximab), radiation doses, and molecular pathological indicators. Second, while PSM aims to mitigate predictable confounding variables, it is unable to balance variables such as patient performance status and comorbidities. Additionally, the matching process resulted in the loss of some samples, which could negatively impact the generalizability of the results. Furthermore, the SEER database does not provide comprehensive details regarding treatment sequences, regimens, or lines of treatment. Consequently, the surgery, chemotherapy, and radiotherapy analyzed in this study pertain to treatments administered during the initial course of therapy, rather than specific first-line or subsequent treatments. This limitation should be considered when interpreting the relationship between treatment modalities and survival outcomes.

Although age and treatment variables are well-documented prognostic factors in lymphoma, the novelty of this study lies not in the identification of entirely new prognostic factors but in its focus on a specific disease context and study design. PPL is a rare condition, and most prior research has relied on small, single-center cohorts or case reports. In contrast, this study utilized a large population-based cohort from the SEER database and employed PSM to minimize baseline differences, facilitating a more balanced comparison of survival outcomes between pulmonary MALT lymphoma and pulmonary DLBCL. Additionally, we developed and internally validated a prognostic nomogram specifically for pulmonary DLBCL, which may offer individualized risk assessments for patients with this rare disease. Thus, this study contributes population-based comparative evidence and a disease-specific prognostic model for PPL. Moreover, while we aimed to identify PPL using SEER primary site and histology codes, the database does not provide adequate clinical details to fully distinguish PPL from systemic lymphoma with secondary pulmonary involvement. Consequently, some cases classified as PPL may actually represent systemic disease affecting the lungs. This limitation is inherent in registry-based studies and should be considered when interpreting the findings. It is important to emphasize that this was an observational study utilizing registry data, and thus, causal relationships between treatments and survival outcomes cannot be definitively established. The observed associations among surgery, chemotherapy, and survival should be interpreted with caution, as treatment selection may be influenced by patient characteristics, disease stage, and other unmeasured confounders.

Future research should encompass several key areas. First, the findings of this population-based study should be validated in prospective cohort studies that include more detailed clinical information, such as treatment intensity, regimens, and molecular subtyping. Second, further investigation is warranted to explore the biological mechanisms underlying the prognostic disparities between pulmonary MALT lymphoma and pulmonary DLBCL, particularly regarding the tumor microenvironment and host immune response. Third, subsequent studies should concentrate on the development and external validation of personalized prognostic models and treatment decision-support systems that integrate multimodal data, including clinical, pathological, molecular, and imaging information. Such approaches may enhance individualized treatment strategies and clinical outcomes for patients with PPL.

## Conclusion

In summary, this paper demonstrates, through extensive population data and robust statistical methodologies, that biological aggressiveness related to pathological subtype is a fundamental determinant of long-term prognosis in PPL, irrespective of conventional staging and baseline treatment. For pulmonary DLBCL, it is essential to address the current limitations of therapy and to devise prognostic methodologies that are not solely based on anatomical staging. This analysis provides vital practical insights and a strategic framework for advancing these objectives.

## Supplemental data

**Figure S1.**
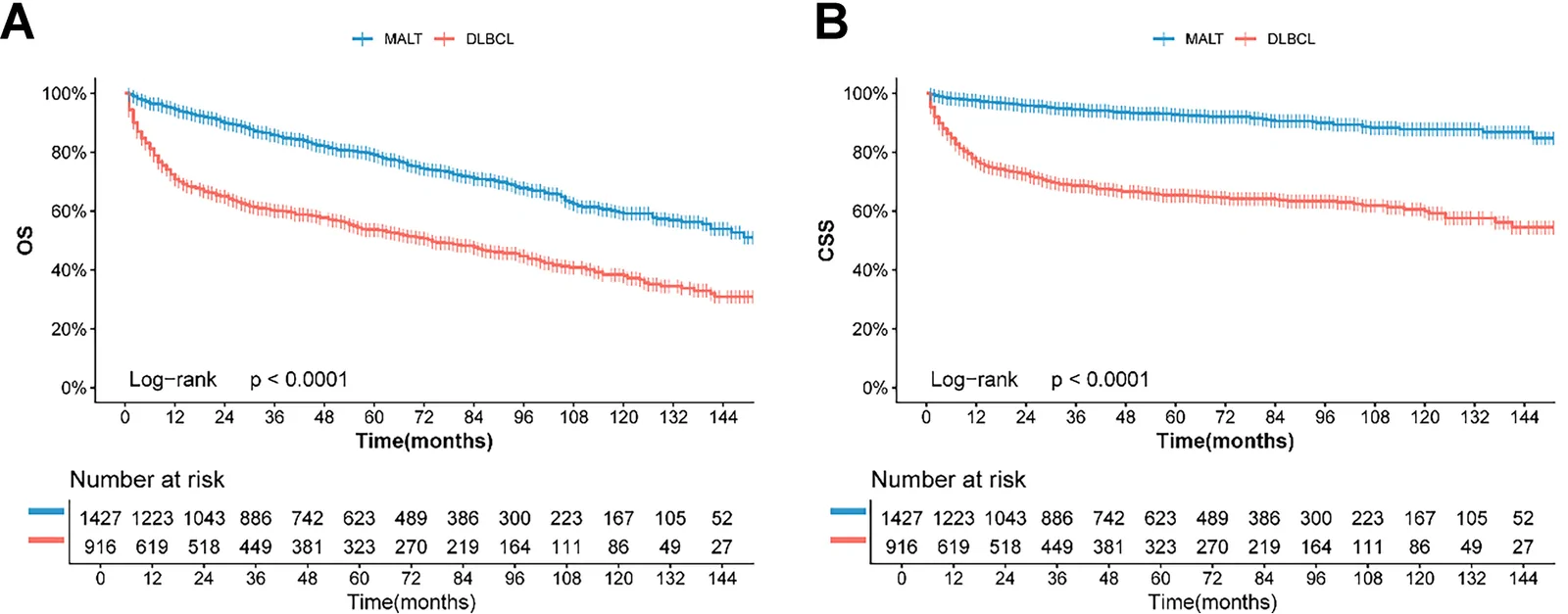
**Kaplan-Meier curves for overall survival and cancer-specific survival before propensity score matching.** (A) Overall survival and (B) cancer-specific survival in the overall cohort, comparing patients with pulmonary mucosa-associated lymphoid tissue lymphoma and pulmonary diffuse large B-cell lymphoma. Before propensity score matching, patients with MALT lymphoma exhibited significantly superior overall survival and cancer-specific survival compared with those with DLBCL (both log-rank *P* < 0.0001). Numbers at risk are shown below the x-axis. **Abbreviations:** CSS, cancer-specific survival; DLBCL, diffuse large B-cell lymphoma; MALT, mucosa-associated lymphoid tissue; OS, overall survival; PSM, propensity score matching.

**Table S1:** Comparison of baseline characteristics of patients

**Variable**	**Overall**	**MALT**	**DLBCL**	***P***	**SMD**
n	2,343	1,427	916		
**Sex, *n* (%)**				0.001	0.138
Male	1,030 (43.96)	589 (41.28)	441 (48.14)		
Female	1,313 (56.04)	838 (58.72)	475 (51.86)		
**Age, *n* (%)**				0.394	0.038
<60	565 (24.11)	335 (23.48)	230 (25.11)		
≥60	1,778 (75.89)	1,092 (76.52)	686 (74.89)		
**Marital, *n* (%)**				0.003	0.145
Unmarried	304 (12.97)	160 (11.21)	144 (15.72)		
Married	1,364 (58.22)	862 (60.41)	502 (54.80)		
SDW	675 (28.81)	405 (28.38)	270 (29.48)		
**Race, *n* (%)**				0.220	0.074
White	1,992 (85.02)	1,208 (84.65)	784 (85.59)		
Black	174 (7.43)	116 (8.13)	58 (6.33)		
Other	177 (7.55)	103 (7.22)	74 (8.08)		
**Multiple cancers, *n* (%)**				0.105	0.071
No	1,494 (63.76)	891 (62.44)	603 (65.83)		
Yes	849 (36.24)	536 (37.56)	313 (34.17)		
**Primary site, *n* (%)**				<0.001	0.284
Main bronchus	54 (2.30)	17 (1.19)	37 (4.04)		
Upper lobe	694 (29.62)	445 (31.18)	249 (27.18)		
Middle lobe	184 (7.85)	136 (9.53)	48 (5.24)		
Lower lobe	705 (30.09)	439 (30.76)	266 (29.04)		
Others	706 (30.13)	390 (27.33)	316 (34.50)		
**AJCC, *n* (%)**				<0.001	0.799
I	1,161 (49.55)	904 (63.35)	257 (28.06)		
II	399 (17.03)	158 (11.07)	241 (26.31)		
III	60 (2.56)	16 (1.12)	44 (4.80)		
IV	629 (26.85)	286 (20.04)	343 (37.45)		
Unknown	94 (4.01)	63 (4.41)	31 (3.38)		
**Surgery, *n* (%)**				<0.001	0.543
No	1,753 (74.82)	945 (66.22)	808 (88.21)		
Yes	590 (25.18)	482 (33.78)	108 (11.79)		
**Radiation, *n* (%)**				0.782	0.014
No/Unknown	2,015 (86.00)	1230 (86.19)	785 (85.70)		
Yes	328 (14.00)	197 (13.81)	131 (14.30)		
**Chemotherapy, *n* (%)**				<0.001	1.340
No/Unknown	1,316 (56.17)	1,112 (77.93)	204 (22.27)		
Yes	1,027 (43.83)	315 (22.07)	712 (77.73)		

**Abbreviations:** AJCC, American Joint Committee on Cancer; DLBCL, diffuse large B-cell lymphoma; MALT, mucosa-associated lymphoid tissue; SDW, separated/divorced/widowed; SMD, standardized mean difference.

## Data Availability

The data used in this study are available from the Surveillance, Epidemiology, and End Results Program (SEER) database (Incidence SEER 17 Registries Nov 2024 Sub) at https://seer.cancer.gov/data-software/documentation/seerstat/nov2024/.
